# Emotionality modulates the impact of chronic stress on memory and neurogenesis in birds

**DOI:** 10.1038/s41598-020-71680-w

**Published:** 2020-09-03

**Authors:** Flore Lormant, Vitor Hugo Bessa Ferreira, Maryse Meurisse, Julie Lemarchand, Paul Constantin, Mélody Morisse, Fabien Cornilleau, Céline Parias, Elodie Chaillou, Aline Bertin, Léa Lansade, Christine Leterrier, Frédéric Lévy, Ludovic Calandreau

**Affiliations:** 1 INRAE, UMR 85 Physiologie de la Reproduction et des Comportements, 37380 Nouzilly, France; 2 CNRS, UMR 7247, 37380 Nouzilly, France; 3 Université François Rabelais, 37041 Tours, France; 4 IFCE, 37380 Nouzilly, France; 5 Yncréa Hauts-de-France, ISA Lille, 48 bd Vauban, 59046 Lille Cedex, France

**Keywords:** Learning and memory, Stress and resilience, Animal behaviour, Behavioural methods, Neurological models

## Abstract

Chronic stress is a strong modulator of cognitive processes, such as learning and memory. There is, however, great within-individual variation in how an animal perceives and reacts to stressors. These differences in coping with stress modulate the development of stress-induced memory alterations. The present study investigated whether and how chronic stress and individual emotionality interrelate and influence memory performances and brain neurogenesis in birds. For that, we used two lines of Japanese quail (*Coturnix japonica*) with divergent emotionality levels. Highly (E+) and less (E−) emotional quail were submitted to chronic unpredictable stress (CUS) for 3 weeks and trained in a spatial task and a discrimination task, a form of cue-based memory. E + and E− birds were also used to assess the impact of CUS and emotionality on neurogenesis within the hippocampus and the striatum. CUS negatively impacted spatial memory, and cell proliferation, and survival in the hippocampus. High emotionality was associated with a decreased hippocampal neurogenesis. CUS improved discrimination performances and favored the differentiation of newborn cells into mature neurons in the striatum, specifically in E+ birds. Our results provide evidence that CUS consequences on memory and neural plasticity depends both on the memory system and individual differences in behavior.

## Introduction

It is now well-accepted that, for humans and non-human animals, there is no single memory system, but multiple memory systems mediating and processing different types of information^1^. This idea came after the convergence of different sources of evidence that impairment in a particular memory task did not mean complete inability to acquire, process, store, and retrieve other types of information^2^. The hippocampus, for example, is responsible for establishing relationships between different cues in the environment and creating a spatial mental map that allows place learning (spatial memory, henceforth). The dorsolateral striatum, in contrast, is specialized in treating cues individually and generating a simpler stimulus–response habit formation (cue-based memory, henceforth)^3–5^.

The way a type of memory is favored over another is modulated by multiple factors, such as stress and its intensity. Acute stress or the injection of anxiogenic drugs may bias rats to preferentially use the striatal-dependent habit memory^6^. Chronic stress, on the other hand, has been repeatedly shown to negatively impact spatial memory in rodents^7,8^, as it profoundly alters hippocampal neurogenesis^9,10^, a multistep mechanism of plasticity that allows the hippocampus to produce and integrate newborn neurons and which is required for proper spatial memory formation and functioning^11,12^. The effects of chronic stress on cue-based memory have been less studied than for spatial memory and present controversial results. Some studies report an absence of stress effects^13^, whereas others report an improvement of cue-based memory abilities^7,14^.

Individual differences are another essential factor to be taken into consideration concerning the vulnerability to develop stress-induced memory alterations^15,16^. The current literature presents results that are not always straightforward. Less exploratory free-range broiler chickens (*Gallus gallus domesticus*), which are known to be more fearful^17^, have better cognitive performance in spatial memory and behavioral flexibility tasks^18–20^. For laying hens, on the other hand, more fearful hens were more predisposed to show side biases than less fearful hens, suggesting that high levels of fearfulness lead to more stimulus–response strategy^21^. As evidenced by^22^, the link between inter-individual differences and cognition is not fully understood and needs further empirical work.

The present study aimed to investigate whether and how chronic stress and individual emotionality interrelate and influence memory performances and brain neurogenesis. For that, we used two unique and valuable lines of Japanese quail (*Coturnix japonica*), divergent in their emotionality levels^23^. These lines were initially selected for their reactions during a tonic immobility (TI) test, a test aimed to measure fearfulness. TI is suggested to have evolved in response to predation: the longer the individual immobility, the higher its fear levels^24,25^. One line has been selected for long TI duration, the other for a short TI duration. Previous studies demonstrated that quail selected for their long TI duration exhibit more fear behaviors when presented to a new environment, a novel object and novel food, compared to quail with shorter TI duration^26–28^. These behavioral differences were attenuated when the animals receive an anxiolytic before being exposed to a new environment^29^.

The first cohort of highly and less emotional birds was submitted to chronic unpredictable stress (CUS) for 3 weeks (stress groups) or left undisturbed (control groups). At the end of the stress procedure, chronically stressed and control birds were submitted, first, to a spatial learning task and then to a discrimination task, a form of cue-based memory. A second, independent cohort of quail was submitted to the same CUS procedure to verify potential effects of stress and emotionality on brain neurogenesis. Using single and double immunohistological labeling of relevant markers of neurogenesis, we measured cell proliferation, cell survival, and neuronal differentiation in both the hippocampus and the striatum of both highly and less emotional birds under control and stress treatments.

The first hypothesis of this study was that the bird hippocampus would be, as for mammals, a structure particularly sensitive to the effects of chronic stress. Thus, chronic stress would specifically induce deficits in spatial memory and hippocampal neurogenesis. On the other hand, stress would have little or no effect on cue-based memory and neurogenesis in the striatum. The second hypothesis was that highly emotional quail would present a greater negative alteration in spatial memory and hippocampal neurogenesis following exposure to chronic stress compared to less emotional quail.

## Results

### Effect of chronic stress on spatial memory

During habituation, the number of mealworms eaten increased over days, confirming that birds habituated to the task (days: F(3,159) = 24.40, *p* < 0.001). Chronic stress had no effect on habituation (stress: F(1,53) = 0.03, *p* = 0.87; emotionality × stress: F(1,53) = 0.78, *p* = 0.38), but highly emotional individuals ate fewer mealworms than less emotional ones (emotionality: F(1,53) = 21.79, *p* < 0.001).

**Figure 1:**
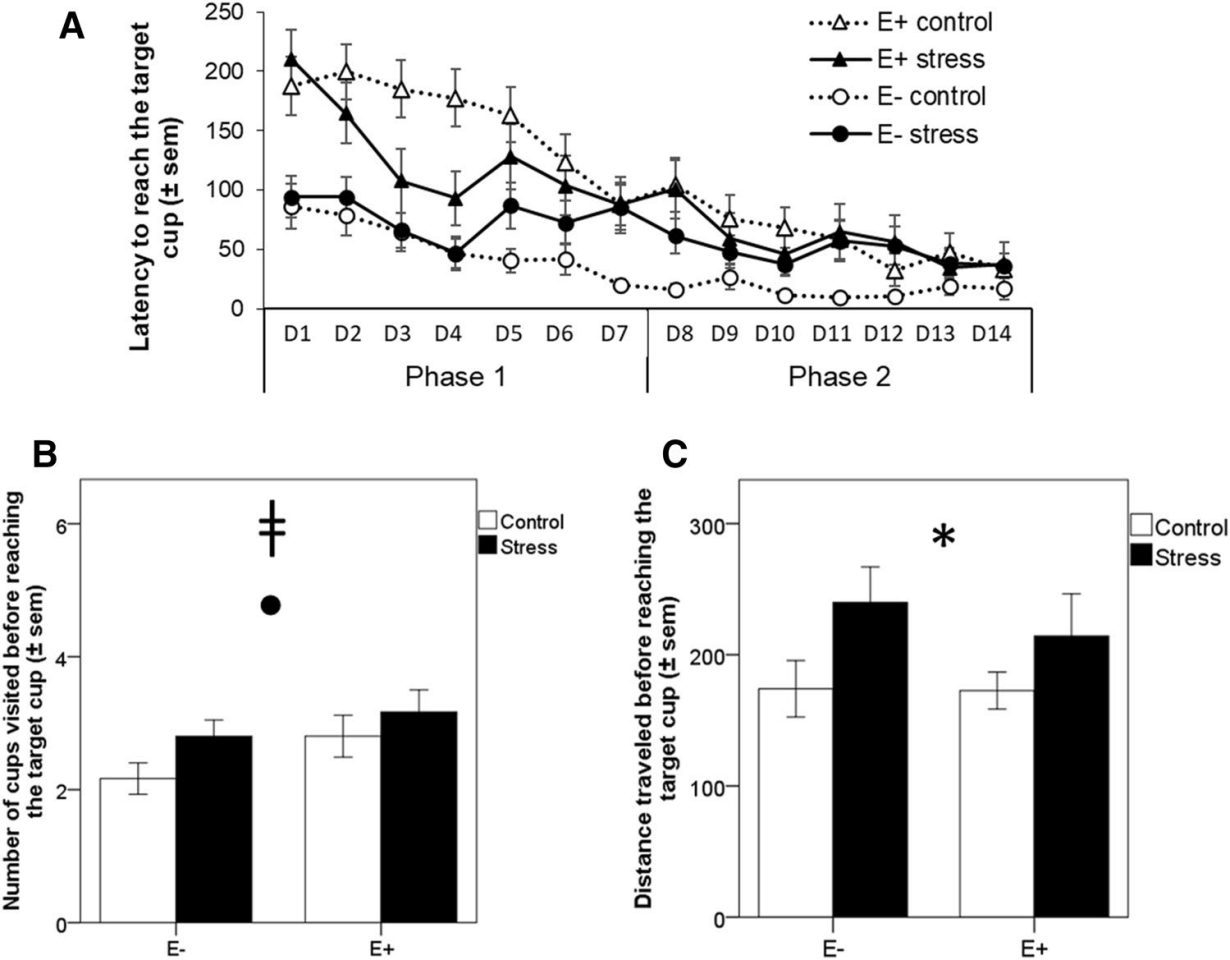
Effect of chronic stress and bird emotionality on spatial learning performances. (**A**) Mean latency (± sem) to reach the location of the target cup over training days for of high (E+) and less (E−) emotional quail submitted (stress) or not (control) to chronic stress. (**B**,**C**) Number of cups visited and distance travelled, respectively, before reaching the target cup (± sem) during the second training phase (from day 8 to 14) in chronically stressed and control E+ and E− birds. ^ǂ^*p* < 0.09, tendency for difference between highly and less emotional quail; Filled circle: *p* < 0.09, tendency for difference between control and chronically stressed quail. *p < 0.05, significant difference between control and chronically stressed quail. Data are presented as mean ± s.e.

During training, the latency to find the unique target cup decreased significantly between training phases (F(1,53) = 81.63, *p* < 0.001, see Table S1). Between the 2 training phases this decrease was significantly different between birds with high and low emotionality levels (training phase × emotionality: F(1,53) = 14.85, *p* < 0.001, Fig. 1A) but not affected by chronic stress (training phase × stress : F(1,53) = 1.95, *p* = 0.17; interaction training phase × emotionality × stress: F(1,53) = 0.71; *p* = 0.40). Post-hoc analyses revealed that highly emotional birds had a significant longer latency to reach the target cup on the first training phase when compared to less emotional ones (*p* < 0.001). In contrast, this difference did not reach significance in the second training phase (*p* = 0.06).

Over phases of training, birds progressively learned to reach the location of the target cup, as the number of visits before reaching the target cup significantly decreased (training phase: F(1,53) = 88.21, *p* < 0.001). Chronic stress and emotionality differentially affected learning according to the training phase (training phase × stress: F(1,53) = 7.53, *p* = 0.008; training phase × emotionality: F(1,53) = 4.14, *p* = 0.047; training phase × stress × emotionality: F(1,53) = 0.337, *p* = 0.53). Post-hoc analyses revealed that while chronic stress did not affect the first training phase (*p* = 0.34), there was a tendency for it to increase the number of visits before reaching the target cup during the second training phase for both highly and less emotional birds (*p* = 0.082, Fig. 1B). High emotionality, on the other hand, increased significantly the number of visits before reaching the target cup in the first training phase (*p* < 0.001), with a tendency for increase over the second training phase (*p* = 0.082, Fig. 1B).

Finally, only chronic stress, but not emotionality nor the interaction between chronic stress and emotionality, increased the individuals’ distance traveled before reaching the target cup (stress: F(1,53) = 4.98, *p* = 0.03; emotionality: F(1,53) = 0.31, *p* = 0.57; stress × emotionality: F(1,53) = 0.25, *p* = 0.61; Fig. 1C).

### Effect of chronic stress on discrimination

During habituation, quail visited more cups and ate significantly more mealworms over days (days: F(3,144) = 46.01, *p* < 0.001; days × emotionality: F(3,144) = 1.30, *p* = 0.28; days × stress: F(3,144) = 1.97, *p* = 0.12). Chronic stress had no effect on habituation (stress: F(1,48) = 0.002, *p* = 0.97; stress × emotionality: F(1,48) = 0.29, *p* = 0.60), but highly emotional birds ate fewer mealworms (emotionality: F(1,48) = 5.54, *p* = 0.02). Moreover, independently of emotionality and chronic stress, quail visited significantly more the white cups than the black cups (color: F(1,48) = 54.05, *p* < 0.0001; color × emotionality F(1,48) = 1.59, *p* = 0.21; color × stress: F(1,48) = 1.63, *p* = 0.43).

**Figure 2:**
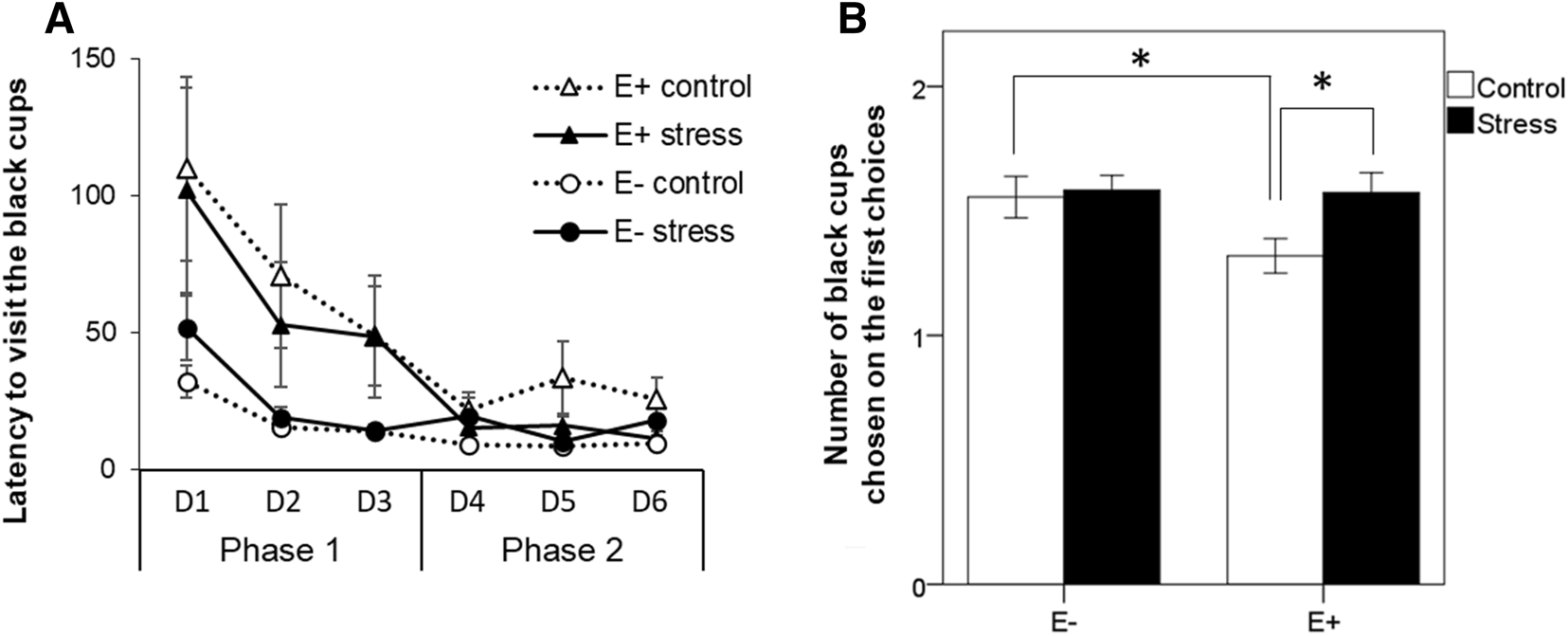
Effect of chronic stress and bird emotionality on discrimination performances. (**A**) Mean latency (± sem) to visit the two target black cups over training days for highly (E+) and less (E−) emotional quail submitted (stress) or not (control) to chronic stress. (**B**) Mean number of black cups (± sem) chosen on the first choices during the second training phase (from day 4 to 6) in chronically stressed and control E+ and E− birds. *p < 0.05. Data are presented as mean ± s.e.

During the training of the discrimination task, similar to what we observed in the spatial task, the latency to reach the two rewarded black cups decreased over the two training phases and this decrease was significantly affected by emotionality (training phase: F(1,48) = 25.95, *p* < 0.001; training phase × emotionality: F(1,48) = 9.90, *p* < 0.01) but not by chronic stress (training phase × stress: F(1,48) = 0.12, *p* = 0.74; training phase × emotionality × stress: F(1,48) = 0.11, *p* = 0.74; Fig. 2A). Post-hoc analyses on each training phase revealed that highly emotional birds had a significant longer latency to reach the target cups during the first training phase (*p* = 0.002) and a tendency for longer latency during the second training phase (*p* = 0.06), compared to less emotional birds.

The number of rewarded black cups visited by quail among their first two visited cups significantly increased between the first and the second training phase (training phase: F(1,48) = 82.40, *p* < 0.001). This increase was dependent on both the emotionality trait and chronic stress (training phase × emotionality × stress: F(1,48) = 5.99, *p* = 0.018). Post-hoc analyses revealed that, during the second training phase, highly emotional chronically-stressed birds were more accurate than highly emotional control birds in selecting the black cups to find food (*p* = 0.035, Fig. 2B). Furthermore, highly emotional control birds exhibited deficits in selecting the black cups during the second training phase compared to less emotional control birds (*p* = 0.026, Fig. 2B).

### Effect of chronic stress in the open-field test

**Figure 3:**
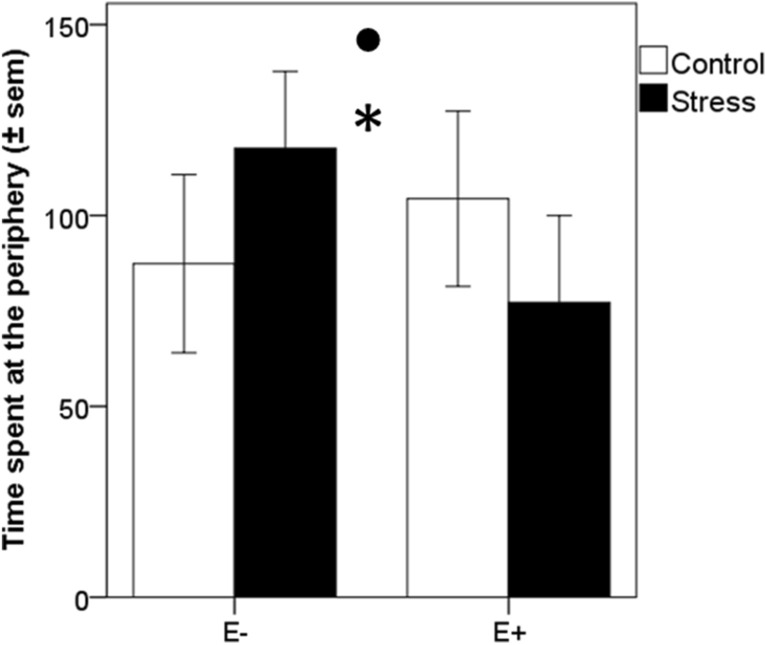
Effect of chronic stress and bird emotionality in the open-field test. (**A**) Mean time spent at the periphery (± sem) of the open field test arena for highly (E+) and less (E−) emotional quail submitted (stress) or not (control) to chronic stress. *p < 0.05, significant difference between control and chronically stressed quail; Filled circle: *p* < 0.05, significant difference between highly and less emotional quail. Data are presented as mean ± s.e.

One week after the end of the discrimination task, reactivity to a new environment was assessed in an open-field test, which was previously reported as a relevant behavioral indicator of chronic stress^27,28,30^. The analysis of the time spent in the inner and outer parts of the open-field arena revealed that the time spent at the periphery of the open field was affected by chronic stress and emotionality. Indeed, both high emotionality (emotionality: F(1,53) = 38.26, *p* < 0.0001) and chronic stress decreased the time spent at the periphery of the arena (stress: F(1,53) = 4.12, *p* = 0.047; emotionality × stress: F(1,53) = 0.13, *p* = 0.72, Fig. 3).

### Effects of chronic stress on hippocampal neurogenesis

**Figure 4:**
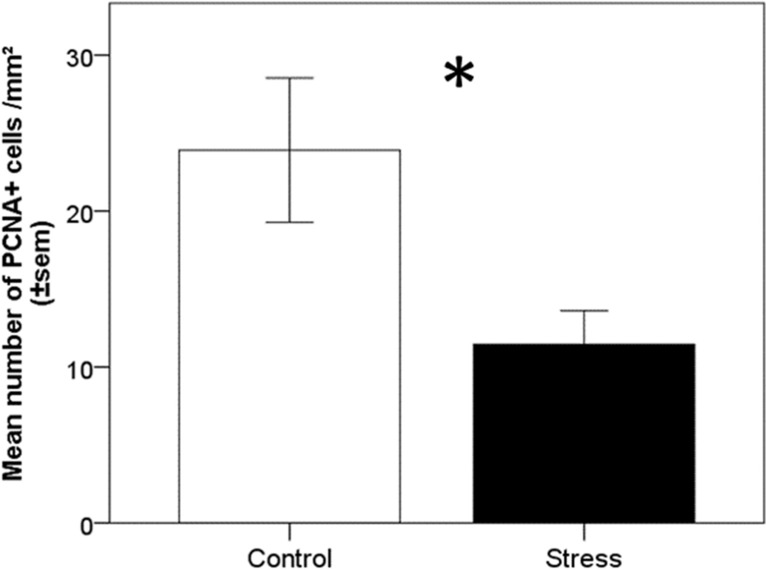
Effect of chronic stress and bird emotionality on hippocampus proliferation. Density of PCNA + cells into the ventricular zone adjacent to the hippocampus of highly (E+) and less (E−) emotional quail, submitted (stress) or not (control) to chronic stress. *p < 0.05. Data are presented as mean ± s.e.

Hippocampal cell proliferation was assessed by measuring the density of PCNA (proliferating cell nuclear antigen) positive cells in the ventricular zone adjacent to the hippocampus (see Table S2). Chronic stress significantly decreased the density of PCNA positive cells (stress: t(1) = − 2.46, *p* = 0.01, Fig. 4), while there were no effects of emotionality (emotionality: t(1) = − 0.757, *p* = 0.45), nor differences between the left and right hippocampus (side: t(1) = − 0.091, *p* = 0.92).

**Figure 5:**
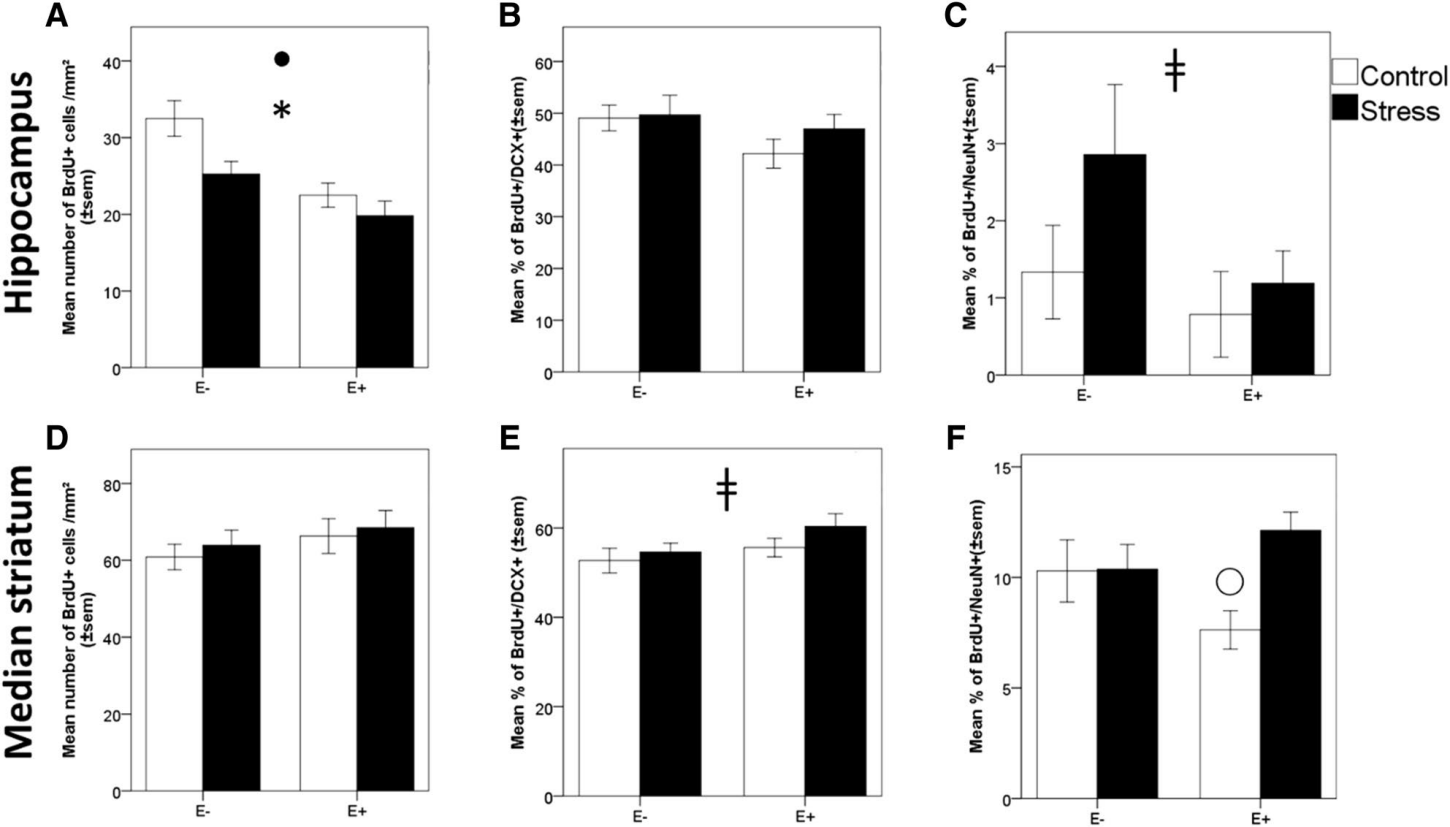
Effect of chronic stress and bird emotionality on hippocampus and striatum neurogenesis. (**A**) Effect of chronic stress on cell survival, (**B**) differentiation of new-born cells in immature and (**C**) mature neurons in the hippocampus of highly (E+) and less (E−) emotional quail, submitted (Stress) or not (Control) to chronic stress. (**D**) Effect of chronic stress on cell survival, (**E**) differentiation of new-born cells in immature and (**F**) mature neurons in the median striatum of highly (E+) and less (E−) emotional quail, submitted (Stress) or not (Control) to chronic stress. *p < 0.05, significant difference between control and chronically stressed quail; Filled circle: *p* < 0.05, significant difference between highly and less emotional quail; ^ǂ^*p* < 0.08, tendency for difference between highly and less emotional quail; Opened circle: *p* < 0.05 for the effect of stress in highly emotional birds. Data are presented as mean ± s.e.

The survival of newborn cells was assessed by measuring the density of BrdU (bromodeoxyuridine) positive cells in two subdivided zones: a ventricular zone and a non-ventricular zone. In the ventricular zone, there was no effect of chronic stress nor emotionality on the density of BrdU positive cells (stress: t(1) = 0.49, *p* = 0.62; emotionality: t(1) = 1.06, *p* = 0.29), however the density was significantly higher on the left side compared to the right side (side: t(1) = − 2.65, *p* = 0.01). In the non-ventricular zone, both chronic stress and high emotionality significantly decreased the density of BrdU positive cells (stress: t(1) = − 2.59, *p* = 0.01; emotionality: t(1) = 4.03, *p* < 0.001, Fig. 5A), with no differences between sides (side: t(1) = − 0.03, *p* = 0.97).

The neuronal differentiation of BrdU positive cells was assessed by using double immunolabeling for BrdU and DCX (doublecortin), a marker of neuroblasts, or for BrdU and NeuN (Neuronal Nuclei), a marker of mature neurons. There were no differences between control and chronic stressed animals in the proportion of BrdU+/DCX+ cells nor BrdU+/NeuN+ cells (stress: t(1) = 0.9, *p* = 0.36 and : t(1) = 1.41, *p* = 0.16, respectively, Fig. 5B). High emotionality did not influence the proportion of BrdU+/DCX+ cells, but tended to decrease the proportion of BrdU+/NeuN+ cells (emotionality: t(1) = 1.59, *p* = 0.11 and : t(1) = 1.75, *p* = 0.08, respectively, Fig. 5C). No significant side differences were found for BrdU+/DCX+ cells nor BrdU+/NeuN+ cells (side: t(1) = 0.4, *p* = 0.68 and : t(1) = 1.64, *p* = 0.10, respectively).

### Effects of chronic stress on neurogenesis in the striatum

In the median striatum, neither chronic stress, emotionality nor side significantly affected the density of PCNA positive cells (stress: t(1) = − 0.98, *p* = 0.33; emotionality: t(1) = − 0.76, *p* = 0.45; side: t(1) = − 0.49, *p* = 0.62) and the density of BrdU positive cells (stress: t(1) = 0.64, *p* = 0.51; emotionality: t(1) = − 1.23, *p* = 0.22; side: t(1) = − 1.61, *p* = 0.11, Fig. 5D). High emotionality tended to increase the proportion of BrdU+/DCX+ cells in the median striatum (emotionality: t(1) = − 1.76, *p* = 0.07, Fig. 5E), but no effect was found for chronic stress or side (stress: t(1) = 1.39, *p* = 0.16; side: t(1) = 0, *p* = 1). Chronic stress differentially affected the proportion of BrdU+/NeuN+ cells in the median striatum, according to emotionality (stress × emotionality: t(1) = − 2.17, *p* = 0.03). Chronic stress significantly increased the proportion of BrdU+/NeuN+ cells in highly emotional birds compared to highly emotional in the control situation (*p* = 0.01; Fig. 5F). No significant differences were found between the right and left sides of the median striatum (t(1) = 0.78, *p* = 0.43).

## Discussion

The present study shows that the effects of chronic stress on memory depend on both the memory system involved and on individual differences in birds’ emotionality. Spatial memory, a form of explicit memory that relies mainly on the hippocampus, appears particularly sensitive to the adverse effect of chronic stress, as it disrupted spatial learning performances similarly for both highly and less emotional birds. On the other hand, cue-based memory, an implicit form of memory relying on the striatum, was shown to be improved by chronic stress depending on birds’ level of emotionality: highly emotional birds had their learning performances increased during the discrimination task. The effects of chronic stress on hippocampal and striatal neurogenesis remarkably mirrored the results observed during our cognitive tasks, as chronic stress disrupted cell proliferation and survival of 3-week newborn cells in the hippocampus. Interestingly, high emotionality was also associated with a decreased cell survival in the hippocampus and with a decreased differentiation of 3-week newborn cells into mature neurons, in the hippocampus. By contrast, neither chronic stress nor emotionality induced detrimental effects on striatal neurogenesis; instead, it favored differentiation of 3-week newborn cells into immature and mature neurons in the medial striatum for the highly emotional birds, similar to its positive effect on cue-based memory.

During the habituation and training phases of the spatial task, highly emotional birds ate fewer worms, had a longer latency and made more visits to other cups before reaching the target cup compared to less emotional birds. These findings may indicate that highly emotional birds were slower to habituate to the apparatus and to learn the task. Although the latency to perform a task is frequently used to measure learning performances, this criterion can be strongly impacted by other factors as the locomotor activity or the emotional state of the subject^31,32^. Thus, higher latencies in highly emotional quail could instead reflect their reluctance to explore unfamiliar environments or objects^27–29^. However, this idea is not supported based on the fact that high emotionality also increased the number of visits before reaching the target cup, a variable that is little impacted by locomotor activity and used to qualify memory performances^8,33^.

During the second training phase, and similar to emotionality, chronic stress tended to increase the number of cups visited and increased the distance travelled before the target cup was reached, showing that chronically stressed birds exhibited a decrease in spatial performance. These results corroborate previous studies in mammals showing that this memory system is negatively impacted by chronic stress^7,8,34^. However, contrary to our hypothesis that highly emotional quail would present a greater negative alteration in spatial memory following exposure to chronic stress compared to less emotional quail, our results indicate that emotionality did not protect from nor exacerbate the adverse effect of chronic stress on spatial memory.

The consequences of chronic stress on birds’ hippocampal neurogenesis, mainly by reducing cell proliferation and survival, closely match their performances in the spatial task, which further corroborates previous works providing evidence for deleterious effects of stressful events on hippocampal neurogenesis^35–37^. In mammals, for example, hippocampal neurogenesis is essential for an optimal spatial memory^11,12^.

Beyond chronic stress, bird emotionality modulated stages of hippocampal neurogenesis such as cell survival and differentiation into mature neurons. These findings suggest that a bird’s hippocampal neurogenesis is not only under the influence of external factors as stressors, learning, and memory experiences^38–40^, but also influenced by intrinsic factors modulating emotional reactivity. In direct line with this, previous studies in mammals demonstrated that a decreased neurogenesis was associated with a more anxious phenotype^41,42^.

Contrary to the spatial task, and confirming our hypothesis, chronic stress did not induce any deficit in the learning of the discrimination task. Furthermore, we observed a positive effect of chronic stress since it improved discrimination performances in highly emotional birds. The impact of chronic stress on forms of cue-based memory is poorly described, and some contradictory results are reported with stress having either a facilitating effect^7,14^ or no effect^13^ on this type of memory. These discrepancies may be related to the fact that the cue-based memory is composed of numerous sub-systems^43^, based on the functioning of different brain areas. Our findings indicate that cue-based memory is less sensitive to the adverse effect of chronic stress and strengthens the idea that explicit memory, like spatial memory, is a specific target of chronic stress^7,8,34^.

An alternative explanation for the absence of a negative impact of the chronic stress on cue-based memory may relate to the fact that the time interval between the end of the stress procedure and the beginning of the discrimination task was longer than that of the spatial task. However, there are two main reasons to rule out this possible confounding effect. The first one is linked to individual behavioral responses in the open field: studies have shown that after stress procedure is over, stressed quail exhibit similar behavioral reactions when tested in the open field only a few days later and when tested 6 weeks later^27,29,30^. Our chronically stressed birds spent less time at the periphery of the open field, a behavior that was previously shown to be a relevant indicator of chronic stress^29^. Indeed, intraperitoneal injections of diazepam, an anxiolytic drug, reduced anxiety in highly emotional birds, as they increased the time spent at the periphery of the open-field. The diazepam effect was selective since it had no effect on other behaviors such as distress calls or escape attempts. These findings revealed that anxiety behaviors, such as freezing, of highly emotional birds in the open field test could be modulated by the administration of an anxiolytic drug^29^. Moreover, we previously showed that chronic stress significantly reduced locomotor activity and decreased the time spent at the periphery (or increased the time spent in the center) of the open field^28^. Collectively the results obtained in the present study confirmed previous studies conducted in Japanese quail by our group: both high emotionality and chronic stress decrease the time spent at the periphery of the open field. The second reason relies on our own neurobiological results: chronic stress did not negatively impact neurogenesis in the striatum. Neither cell proliferation along the ventricular zone, nor cell survival, nor differentiation into immature and mature neurons was disrupted in the striatum. By contrast, chronic stress enhanced differentiation into immature and mature neurons in the striatum of highly emotional birds, which may explain their improved performances on the discrimination task.

Whereas highly emotional, stressed birds showed improved performances in the discrimination task, highly emotional control birds exhibited deficits in learning it. This finding may indicate that high emotionality negatively impacts cue-based memory performances. Nevertheless, this interpretation does not explain why chronic stress favors cue-based memory performances in stressed emotional birds. In previous studies it was shown that, during a dual spatial/cued probe test, highly emotional quail relied mainly in their spatial memory than their cue-based memory system^5,44^, if that was the case for our birds, such predominance of the spatial memory system may have resulted in lower performances when they were submitted to the cue-based, discrimination task. Such predominance of the spatial memory system may also have been induced by the fact that birds were trained to solve a spatial task before the discrimination task. By impairing spatial memory, chronic stress would then facilitate the use of a cue-based memory system resulting in better performances in the discrimination task, similar to what was reported in mammals^1,45,46^. If that is true, it could suggest that emotionality levels influence the use or interactions between memory systems.

Finally, our data also show that the density of BrdU positive cells was higher on the left side compared to the right side, which suggests that some mechanisms of plasticity may be lateralized in the quail or the bird’s hippocampus. Further research is still needed to fully understand these specificities of the bird’s hippocampus, however, our results are in line with this previous studies that provided evidence for a different role of the right and left hippocampus in learning and memory in birds^47,48^. Recent studies also showed a functional specialization in birds along the rostro caudal hippocampal axis^35^. Together these findings highlight the relevance to study mechanisms of brain plasticity in other species than mammals.

In conclusion, the present study provides evidence that the consequences of chronic stress on memory and neural plasticity depends crucially on the memory system or the brain area considered, the strong parallel between the effects of chronic stress on spatial memory with those observed on hippocampal neurogenesis and cue-based memory and striatum neurogenesis support this idea. Finally, our study emphasizes the importance of taking individual differences into account as they may modulate the effect of chronic stress on memory systems and neurogenesis.

## Materials and methods

### Animals

Two lines of Japanese quail, high and low in emotionality (E+ and E−, respectively), were used. These lines were selected and bred at the INRA poultry experimental facilities (INRA 1295 Unité Expérimentale Pôle Expérimental Avicole de Tours, F-37380 Nouzilly, France), where the experiment took place. On the day of hatching, chicks from the same line were transferred to communal floor pens. At 21 days-old chicks were sexed, and males were reared in single home cages (41 × 51 × 25 cm) in a battery system under a 12:12 h light–dark schedule (light on 08 h). All experiments were conducted in male quail. A piece of artificial turf was placed on the cage floor to allow for dust-bathing behaviors. Ambient temperature was maintained at approximately 20 ± 2 °C. Unless otherwise specified, food and water were provided ad libitum.

Animal care and experimental treatments complied with the guidelines of the French Ministry of Agriculture for animal experimentation and European regulations on animal experimentation (86/609/EEC). They were performed following the local animal regulation (authorized C37-175-1) of the French Ministry of Agriculture under the EEC directive and under ethics committee approval (Val de Loire, agreement N° 1789 and 1848).

### Chronic stress procedure

When male quail were transferred in individual cages (3 weeks-old), they were divided into stress and control groups. In the stress group, quail were submitted to chronic unpredictable stress (CUS) for 21 days. The procedure of CUS consisted of five to six negative stimulations or stressors per day during both night and day. Each negative stimulation occurred at unpredictable times each day to improve the unpredictability of the stressor delivery and decrease habituation to the stress procedure. Briefly, some stressors were delivered directly in the home cage of the quail: confinement of the bird in the corner of its home cage for 30 min, food frustration by placing transparent devices on the feeder just before light on for 30 min, soft home cage shaking (2 × 15 min interspaced by 15 min), unexpected sounds (100 dB) composed of different sounds having or not biological significance for quail. Disturbances from outside the home cage were also delivered, such as a rapid passage of a plastic stick on the rods of the home cage twice a day during 2 min, fast sprayings of water or air on feathers (2 sprays interspaced by 2 min), waving of a plastic flag in front of the home cage for 2 min. Other stressors were also delivered out of the home cage when birds were transferred individually into a new environment for 30 min. Birds from the same line were also placed together in a transport cage for 30 min or placed on a cart and rolled about in the facility.

Control groups were left undisturbed except for routine husbandry procedures similar to that provided to birds exposed to CUS. They received a similar time of visit from the experimenter in the room to control for the time spent in the animal room. Moreover, systematically, when a stressor prevented the bird from having access to the food at the CUS group, an opaque device was also placed on the feeder of control birds to prevent them from eating food for an equivalent time.

### Cognitive and behavioral tests

**Figure 6:**
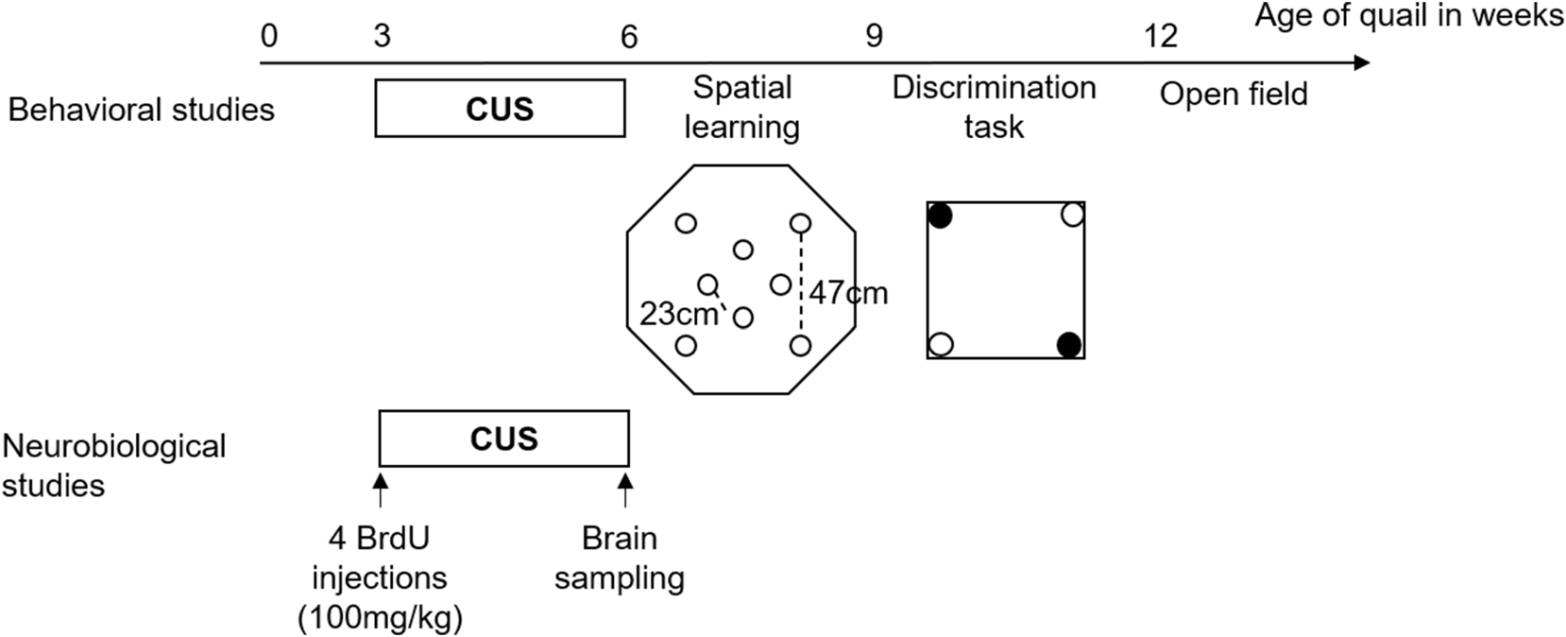
Time schedule of the experiment. At the age of 3 weeks, highly and less emotional quail were submitted to chronic unpredictable stress (CUS) during 3 weeks. At the end of the CUS procedure, a first cohort of animals was submitted to a spatial learning task followed by a discrimination task, and an open field test. A second and independent cohort of quail received 4 intraperitoneal BrdU injections, was submitted to the CUS procedure and used for the study of neurogenesis.

At the end of the CUS procedure, when birds were 6 weeks old, and in order to determine the effect of chronic stress on different memory systems in relation with emotionality level, 15 E− control, 16 E− stress birds, 15 E+ control and 11 E+ stress birds were submitted to two learning tasks (spatial learning and discrimination tasks) and a behavioral test (open field). During the spatial learning task, birds had to learn the spatial location of a target cup among eight identical cups placed in an arena. In the discrimination task, birds had to learn to discriminate between two rewarded black cups and two empty white cups. At the end of the experiment, an open-field test was used to assess the state of stress of birds. The general schedule for behavioral studies is described in Fig. 6. All behavioral data were recorded by a camera placed above the apparatus and computerized by a tracking video system (Ethovision XT; Noldus IT, The Netherlands).

#### Spatial learning task

The following procedures and the arena used for the spatial learning task were previously used and validated by our group^5,44^. In particular, using the exact same procedure in Japanese quail we showed that this spatial learning task requires the functional integrity of the hippocampus in Japanese quail^5^.

##### Habituation

Each bird was individually placed in an octagonal arena (120 cm largest diameter; 50 cm side length) surrounded by a blue curtain (1.90 m high) to prevent the animal from escaping. A beige linoleum covered the floor of the arena, and the arena was lighted by a bulb at the ceiling (18 lx). Four black visual cues were placed on the walls of the arena and four others on the curtain. Eight identical opaque ceramic cups were placed in the arena^5,44^.

Before each habituation session (~ 1 h), food was removed from the quail’s home cage. Once a day, and for 4 days, each quail was transported to the testing room and introduced into the center of the arena. All cups within the arena contained a mealworm. The mealworms were visible in the cups only from a short distance; therefore, the birds needed to approach the cups one by one to see their contents. The bird could freely explore the arena until all cups were visited and all mealworms eaten or for a maximum time of 600 s. On each day, the number of worms eaten was scored^5,44^.

##### Training

Over 2 weeks (14 days), birds were submitted to two spatial training trials per day, spaced by an hour. On each trial, the individual had to reach the location of the only target cup (3–4 mealworms). The other seven cups were identical to the target cup but did not contain any reward. The location of the target cup was constant across all days and trials, and it was the same for all individuals. The tested bird was introduced into the arena by three different starting points. This starting point was randomly chosen among trials. The trial ended when the tested bird reached the target cup and ate the mealworms or after a maximum time of 300 s, whichever came first. In order to keep the individual motivated, when it was not able to reach the target cup, the experimenter gently guided the animal to the target cup and allowed it to eat the mealworms, before removing it from the arena. Between each trial, the bird returned to its home cage^5,44^.

On each trial, the number of cups visited before reaching the target cup was recorded. If the target cup was not visited, a score of eight, corresponding to the total number of cups in the arena, was assigned. The latency to reach the target cup was also measured. As per the number of cups visited before reaching the target cup, if the target cup was not visited the maximum latency (300 s) was assigned. Finally, the distance traveled (in cm) before reaching the target cup was also measured, as we could not assign a maximum distance for individuals that did not reach the cup, this variable was analyzed only from the last 7 days of training, when all individuals could find the cup within the time allotted (see “Statistical analysis” section).

#### Discrimination task

##### Habituation

Four days following the end of spatial learning, habituation to the discrimination task started (by the ninth week of life). During 4 days, once a day, quail were individually transported in an opaque transport box and manually introduced into the center of a white square arena (80 L × 80 l × 80 h cm). A beige linoleum covered the floor of the arena. The arena was surrounded by a green curtain (1.90 m high) and lighted by a bulb at the ceiling (20 lx). Four cups were placed in each corner of the arena: two white cups were placed on one diagonal of the arena, and two black cups were placed in the other diagonal. In each habituation day, the four cups were rewarded, and birds were allowed to freely explore the arena and the cups until they found and eat all mealworms or after a maximum time of 600 s. Similar to the spatial learning task, the mealworms were visible in the cups only from a short distance; therefore, the birds needed to approach the cups one by one to see their contents. On each day, the number of mealworms eaten was scored.

##### Training

Because during habituation quail visited significantly more the white than the black cups (see “Results” section), during six consecutive training days, the two black cups were systematically rewarded and contained a mealworm, whereas the two white cups were never rewarded. This procedure was used to ensure that the birds successfully performed the discrimination task on the basis of learning and not on the basis of a spontaneous preference.

To learn the task, birds needed to discriminate between the black (rewarded) and white cups. Birds were individually submitted to 2 training trials per day with an inter-trial interval of 1 h. In order to ensure that birds did not use the spatial location of the cups to find mealworms in the arena and to force them to learn the task based on the color of the target cup, between each trial, the diagonal composed of white cups and the one composed of black cups were interchanged. Between each trial, birds returned into their home cage.

Each trial ended when birds found the two target cups and eat the mealworms, or after a maximum time of 300 s. On each trial, the latency to reach the target cups and eat all mealworms, and the number of black cups on the first two choices was scored (0, 1, or 2 black cups visited first).

### Open field test

One week after the end of the discrimination task (by the twelfth week of life), reactivity to a new environment was assessed in a square arena (80 L × 80 l × 80 h cm) of white wood with a floor made of beige linoleum. The arena was placed into an unknown experimental room, surrounded by an unknown white curtain and a very bright (50 lx) light illuminated the area. Each quail was individually placed into the center of the arena and allowed to explore it for 5 min freely. On each trial, the time spent in the inner area (a square area of 20 cm × 20 cm at the center of the arena) and at the periphery (band of 40 cm wide from the outer of the arena) of the arena was recorded.

### Immunohistochemistry

Since previous studies demonstrated that a learning task or physical activity can itself influence the neurogenesis^39,49^, and to limit these possible interferences and specifically study the impact of chronic stress on neurogenesis, another independent cohort of birds was used, and their brains were sampled at the end of the CUS procedure (Fig. 6).

#### BrdU injections and tissue preparation

To assess cell survival and fate, the first day of the stress procedure, birds received four intraperitoneal injections of BrdU 2 h apart (100 mg/kg in 0.9% saline; Sigma-Aldrich, Saint- Quentin Fallavier, France), a thymidine analog that is incorporated into the DNA during the S-phase of mitotic division. The dose and procedure were adapted from previous studies demonstrating multiple administrations of a dose of 100 mg/kg was successful to study neurogenesis in birds^50,51^.

At the end of the stress procedure (3 weeks after BrdU injections), birds were anesthetized with an overdose of pentobarbital 6% (36 mg/ 100 g) and were transcardially perfused with 80 mL 1% sodium nitrite in phosphate-buffered saline, followed by 300 mL of ice-cold 4% paraformaldehyde solution in 0.1 M phosphate buffer containing 15% (v/v) saturated picric acid. At the end of the perfusion, brains were removed from the skull, post-fixed overnight in the same fixative, and cryoprotected in a 20% sucrose solution. Coronal free-floating sections were cut on a cryostat at a thickness of 30 µm and stored in Tris-buffer saline 0.025 M, pH 7.4, sodium azide 0.1% (TBSA). Immunohistochemistry was processed on eight brains per group except for the PCNA study for which five brains were sampled.

#### Cell proliferation

To assess cell proliferation in the ventricular zone in birds, we used immunolabeling for the endogenous marker Proliferative Cell Nuclear Antigen (PCNA) that was already validated in previous studies^51,52^. To increase tissue permeabilization, sections were first treated in methanol 100% for 30 min at 30 °C. After four rinses in Tris-buffer saline 0.025 M, pH 7.4 (TBS), sections were treated in TBSA-T (Triton 0.3%)-BSA (Bovine Serum Albumin) 1% for 1 h at room temperature to increase the permeability of plasma membrane. Then, sections were incubated overnight at 4 °C in 1:1,000 anti-PCNA primary antibody (mouse anti-PCNA clone PC10; Millipore, St. Quentin-en-Yvelines, France) in TBSA-T-BSA 1%. On the following day, sections were rinsed four times in TBS-T before being incubated for 2 h at 4 °C in 1:1,000 secondary antibody (donkey anti-mouse CY3; Jackson Immunoresearch, Peterborough, United Kingdom) in TBS-T-BSA 0.1%. After several rinses in TBS, sections were immersed in Hoechst (nuclear staining) for 2 min (Hoechst 33258, 2 µg/mL in distilled water, Invitrogen, Cergy Pontoise, France) and rinsed four times in TBS. All sections were finally mounted on gelatin-coated glass slides, dried and covered by using Fluoromount-G (SouthernBiotech, USA) and stored at 4 °C in the dark.

#### Cell survival

To assess cell survival in the hippocampus and the striatum, we used single immunolabeling to reveal BrdU positive cells. First, sections were treated in TBSA-T-BSA 1% for 1 h at room temperature. Then sections were treated in 2 N HCl in TBS at 37 °C for 30 min to denature the DNA. After four rinses in TBS, sections were incubated overnight at 4 °C in 1:300 anti-BrdU primary antibody (rat anti-BrdU, AbCys S.A., Paris, France) in TBSA-T-BSA 1%. On the following day, sections were rinsed four times in TBS-T and were incubated for 2 h at 4 °C in 1:1,000 secondary antibody (donkey anti-rat CY3 for the hippocampus; donkey anti-rat Alexa fluor 488 for the striatum; Jackson Immunoresearch, Peterborough, United Kingdom) in TBS-T-BSA 0.1%. Sections were finally rinsed in TBS before being mounted on gelatin-coated glass slides, dried and covered by using Fluoromount-G (SouthernBiotech, USA) and stored at 4 °C in the dark.

#### Neuronal differentiation of newborn cells

To characterize the phenotype of BrdU-positive cells in both the hippocampus and the striatum, two different markers were used: DCX (for proliferating and migrating neuroblasts:^53,54^) and NeuN (for post-mitotic neurons:^55,56^). Double-Immunofluorescence labeling was performed against BrdU and each of the two markers. To protect neuronal antigens (DCX or NeuN) against deterioration by HCl treatment used for BrdU staining, immunolabeling for neuronal markers were first conducted, then followed by a BrdU immunolabeling as described above except for the first treatment in TBSA-T-BSA 1% that was not performed.

For DCX labeling, sections were first treated in 0.1% NaBH_4_ in TBS for 30 min at room temperature. After four washes in TBS, sections were treated for 1 h at ambient temperature in TBSA-T-horse serum 2.5% and incubated in 1:150 goat anti-DCX antibody (polyclonal IgG Goat anti-DCX Santa Cruz ref sc-8066, Santa Cruz Biotechnology, Tebu-Bio, Le Perray en Yvelines, France) in TBSA-T-horse serum 2.5% for 48 h at 4 °C. Then sections were rinsed four times in TBS-T and incubated for 2 h at 4 °C in 1:200 secondary antibody (Donkey anti-goat CY3, Jackson Immunoresearch, Peterborough, United Kingdom) in TBS-T-horse serum 2.5%. Sections were rinsed four times in TBS, immersed 15 min in 4% paraformaldehyde solution in 0.1 M phosphate buffer at room temperature to protect DCX labeling from HCl deterioration, and then rinsed four times in TBS. BrdU immunolabeling was then conducted as described above before except that the secondary donkey anti-rat Alexa fluor 488 was diluted in TBS-T-horse serum 2.5%.

For NeuN labeling, sections were first treated in methanol for 30 min at 30 °C. After four washes in TBS, sections were treated for 1 h at ambient temperature in TBSA-T-BSA 1%, followed by overnight incubation at 4 °C in 1:500 mouse anti-NeuN primary antibody (clone A60, Millipore, St. Quentin-en-Yvelines, France) in TBSA-T-BSA 1%. The following day, sections were rinsed four times in TBS-T and incubated 2 h at 4 °C in 1:1,000 secondary antibody (Donkey anti-mouse CY3, Jackson Immunoresearch, Peterborough, United Kingdom) in TBS-T-BSA 0.1%. Sections were rinsed in TBS before starting BrdU labeling.

At the end of all immunohistological labelings, all sections were mounted on gelatin-coated glass slides, dried, coverslipped under Fluoromount-G (SouthernBiotech, USA), and stored at 4 °C in the dark.

#### Immunostaining analysis

**Figure 7:**
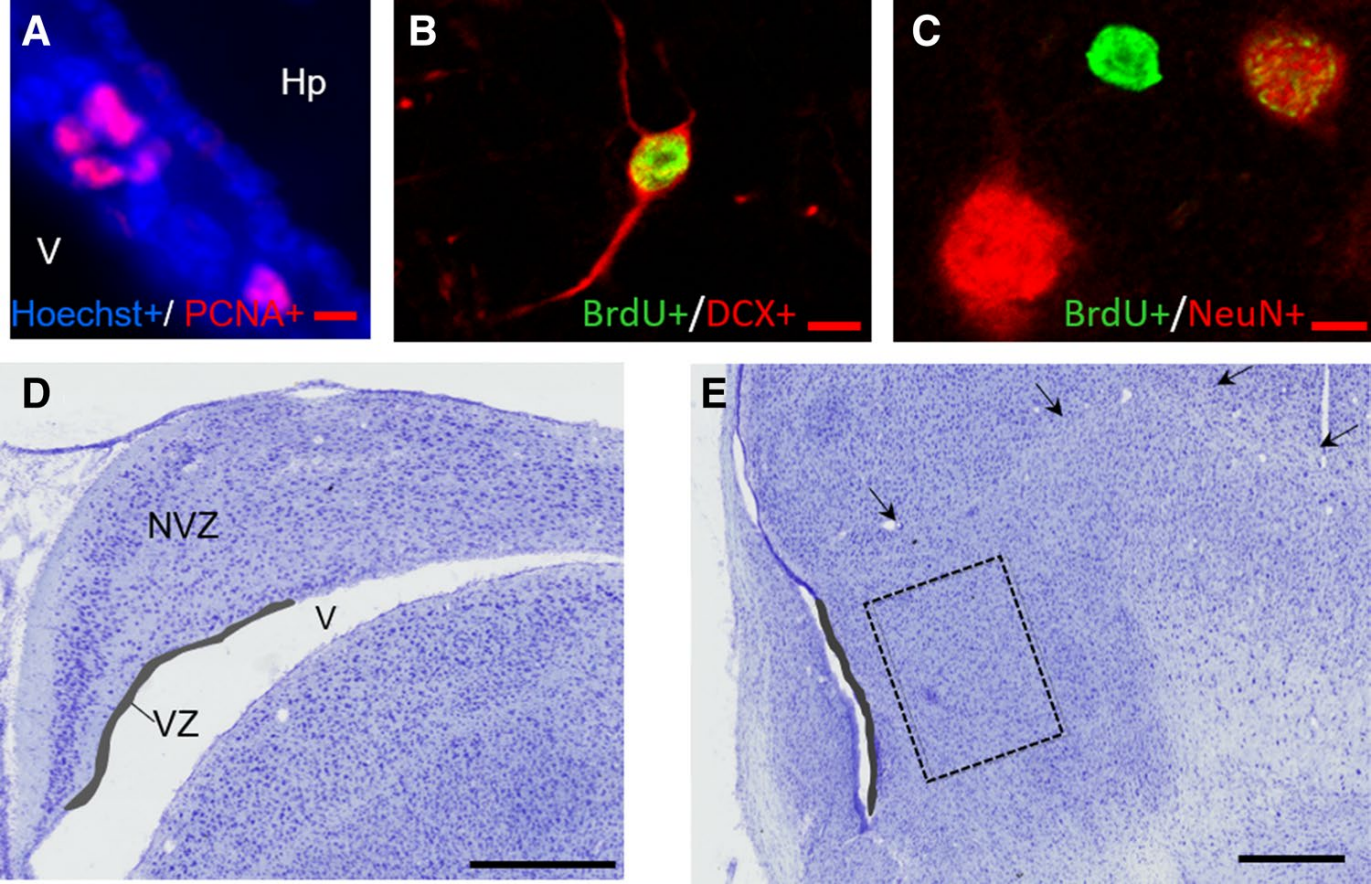
Illustration of staining and counted zones used for hippocampus and striatum neurogenesis. (**A**) Example of immunostaining of PCNA in the ventricular zone. (**B**) Confocal image depicting colocalization of BrdU+/DCX+ (**C**) and BrdU+/NeuN+. (**D**) Cresyl violet-stained frontal sections showing the hippocampus and its subdivision into the ventricular and non-ventricular zone and (**E**) illustration of the rectangle used as the counting area in the median striatum (*V* ventricle, *Hp* hippocampus, *VZ* ventricular-zone, *NVZ* non-ventricular zone; arrowheads, striatum outline; scale bar: (**A**–**C**): 5 µm; (**D**,**E**): 500 µm).

The number of PCNA or BrdU positive cells was manually counted with a fluorescence microscope (Axioskope 2; Zeiss, Germany) at a magnification of × 20 associated with a cell count analysis software (Mercator v7.13.3; Explora Nova, La Rochelle, France) through a black and white camera. The number of PCNA positive cells (Fig. 7A) was counted on average 5–6 sections per animal in the ventricular zone (25–30 µm wide) adjacent to the hippocampus (from A3.0 to 5.5) and to the striatum (from A6.0 to 8.0 according to the quail brain atlas^57^). The area of the ventricular zone was measured on each section with the Mercator software by outlining. The density of PCNA positive cells was averaged (total number of cells divided by the total area of all sections) to obtain a single value per brain.

The number of BrdU positive cells was counted in 4 sections per animal for the hippocampus (from A3.5 to 5.5) and the striatum (from A6.5 to 8.0). The area of the hippocampus was measured with the Mercator software by outlining the entire structure and subdivided into a ventricular zone (VZ) and a non-ventricular zone (NVZ; Fig. 7D). The thickness of the area bordering the ventricle (VZ) was of 25–30 µm and its length varied according to the antero-posterior level of the sections examined (mean length for the hippocampus 1.5 mm; for the striatum 1 mm). In the striatum, BrdU positive cells were counted in a rectangle (700 × 900 µm) positioned into the medial striatum, 200 µm laterally from the ventricle (Fig. 7E). The density of BrdU positive cells was averaged by dividing the total number of positive cells by the total area of the counted region.

For the counting of BrdU and PCNA, subsequent 4–5 sections/animal were analyzed, with 450 µm between sections. Quantification was done on both hemispheres. A scalpel mark was made in the right hemisphere of each brain. For the manual counting of PCNA and BrdU immunopositive cells we adjusted brightness and contrast to have the best discrimination between the cells and the background. We kept the same settings for all sections. Only white and round or ovoid stains were counted as positive nuclei. We could check that the selected object was a cell using the counterstaining with Hoechst. All the structures were counted by one observer blind to experimental conditions.

To determine the percentage of BrdU+ cells that also expressed a neuronal marker (DCX or NeuN, Fig. 7B,C), a confocal laser-scanning microscope (LSM 700; Zeiss; Germany) was used. Each BrdU positive cell was analyzed in its entire z-axis with 0.5 µm step intervals through an × 40 oil immersion objective to identify double labeling. For double labeling in the hippocampus (A4.0 to A5.5) and the striatum (A6.5 and A8.0), at least 50 BrdU positive cells were analyzed per animal. The percentage of double-labeled cells was calculated as [(BrdU+/DCX+ or NeuN+ cell)/(total BrdU+ counted cells)] × 100.

### Statistical analysis

Our response variables were as follows: the number of mealworms eaten during habituation, for both spatial and discrimination tasks. The latency to find the target cup, and the number of cups visited before reaching the target cup, for the spatial task. The latency to find the target cups, and the number of black cups on the first two choices, for the discrimination task. All variables were analyzed by parametric analyses of variance (ANOVA) with emotionality (E+ and E− individuals), groups (stress and control) and cup color (only for the habituation of the discrimination task) as between-subject factors and days (mean values for both training trials within days) as within-subject factors. For the training period, instead of days, two training phases, split into two equal-length phases, consisting of the 7 or 3 days (mean values for each phase), for the spatial and discrimination tasks, respectively, were used as within-subject factors. The distance traveled (in cm) before reaching the target cup was analyzed only for the second training phase, when all individuals could find the cup within the time allotted. This was done since no maximum distance could be assigned, and some individuals needed to be guided to the target cup over the first days of the first training phase.

Data from the open-field were also analyzed through an ANOVA with emotional lines and groups as between-subject factors.

3 E+ control and 2 E+ stressed birds failed to explore the apparatus for more than 3 days in the discrimination task; these birds were thus were excluded for the analyses. Our final n was comprised of 15 E− control, 16 E− stressed birds, 15 E+ control and 11 E+ stressed birds for the spatial memory task, and of 15 E− control, 16 E− stressed birds, 12 E+ control and 9 E+ stressed birds for the discrimination task.

As parts of the bird brain are lateralized^58^, such as for the hippocampal function^59^, PCNA and BrdU densities and percentage of double-labeling were analyzed in the right and the left hemisphere. To investigate the factors affecting neurogenesis, the data were rounded to the nearest whole number and Generalized linear models (GLM) with a Poisson error structure and logarithmic link were used. Since the Poisson GLMs were over dispersed in all models generated, the analyses were repeated using a GLM with a quasi-Poisson error structure. Emotionality (E+ and E− individuals), groups (stress and control) and side (right and left), as well as their interactions were included in the first models. Contrary to the ANOVA, where the main effects can be regarded without the confounding effect of the interaction term (i.e. without the need of removing the interaction, as per the principle of marginality), here, the interactions that were not significant were excluded from the final models. For all analyses, when main effects or interactions were significant, analyses were followed by multiple comparisons corrected by Bonferroni.

All statistical analyses were performed using IBM SPSS 21 and R v. 3.6.1. Statistical significance was accepted at *p* ≤ 0.05 and tendency at *p* < 0.09.

## Supplementary information

Supplementary Information.

Supplementary Tables.
