# Supplementary material for: Emotionality modulates the impact of chronic stress on memory and neurogenesis in birds
Source: Sci Rep. 2020 Sep 3;10:14620. doi: 10.1038/s41598-020-71680-w (PMC7471904; doi:10.1038/s41598-020-71680-w)
Supplement: Supplementary file 2 — Supplementary Tables. [file 41598_2020_71680_MOESM2_ESM.pdf]

Emotionality modulates the impact of chronic stress on memory and neurogenesis in birds

Flore Lormant[1*], Vitor Hugo Bessa Ferreira[1, 2*], Maryse Meurisse[1], Julie Lemarchand[1], Paul Constantin[1], Mélody Morisse[1], Fabien Cornilleau[1], Céline Parias[1], Elodie Chaillou[1], Aline Bertin[1], Léa Lansade[1], Christine Leterrier[1], Frédéric Lévy[1], Ludovic Calandreau[1]

S1 Table. Results for spatial and discrimination memory tasks, open-field behavior and associated statistics

Test	E-		E+		Statistics and p-values			
	Control	Stress	Control	Stress	Days/Phase	E+ vs E-	Stress	Interaction
**Spatial memory task**								
Number of mealworms eaten (Habituation)	7,13 ± 0,73	6,87 ± 1,06	5,15 ± 1,82	5,52 ± 1,52	**F(3,159)=24.40, p<0.001**	**F(1,53)=21.79, p<0.001**	F(1,53)=0.03, p=0.87	NS
Latency to reach the target cup (Training - First phase)	54 ± 45,05	78,09 ± 53,36	160,74 ± 87,19	127,88 ± 82,37	**F(1,53)=81.63, p<0.001**	**F(1,53)=12.55, p=0.001**	F(1,53)=0.16, p=0.742	**Training phase x Emotionality: F(1,53)=14.85, p < 0.001**
Latency to reach the target cup (Training - Second phase)	15,83 ± 13,33	47,33 ± 45,11	60,14 ± 59,85	57,03 ± 79,45				
Number of visits before reaching the target cup (Training - First phase)	3,5 ± 0,65	3,53 ± 0,96	4,88 ± 1,22	4,31 ± 1,43	**F(1,53)=88.21, p<0.001**	**F(1,53)=10.07, p=0.003**	F(1,53)=0.33, p=0.53	**Training phase x emotionality: F(1,53)=4.14, p=0.047**
Number of visits before reaching the target cup (Training- Second phase)	2,16 ± 0,91	2,80 ± 0,97	2,8 ± 1,22	3,16 ± 1,1				**Training phase x Stress: F(1,53)=7.53, p=0.008**
Distance traveled before reaching the target cup (cm) (Training - Second phase)	174,08 ± 83,5	239,94 ± 108,18	172,63 ± 54,69	214,33 ± 106,61	/	F(1,53)=0.31, p=0.57	**F(1,53)=4.98, p=0.03**	NS
**Discrimination task**								
Number of mealworms eaten (Habituation)	1,78 ± 0,2	1,7 ± 0,28	1,45 ± 0,54	1,51 ± 0,55	**F(3,144)=46.01, p<0.001**	**F(1,48)=5.54, p=0.02**	F(1,48)=0, p=0.97	NS
Latency to reach the target cups (Training - First phase)	22,55 ± 10,97	29,43 ± 20,74	78,86 ± 80,41	75,55 ± 89,4	**F(1,48)=25.95, p<0.001**	**F(1,48)=10.58, p=0.02**	F(1,48)=0, p=0.95	**Training phase x Emotionality: F(1,48)=9.90, p<0.01**
Latency to reach the target cups (Training- Second phase)	9,28 ± 2,99	16.07 ± 12,6	27,11 ± 27,42	14,61 ± 6,63				
Number of rewarded black cups visited (first choices) (Training - First phase)	1,13 ± 0,2	1,22 ± 0,25	1,11 ± 0,21	1,01 ± 0,42	**F(1,48)=82.40, p<0.001**	F(1,48)=3.6, p=0.064	F(1,48)=1.28, p=0.26	**Training phase x Emotionality x Stress: F(1,48)=5.99, p=0.018**
Number of rewarded black cups visited (first choices) (Training- Second phase)	1,55 ± 0,32	1,58 ± 0,23	1,31 ± 0,24	1,57 ± 0,23				
**Open field**								
Time spent at the periphery of the arena	169,17 ± 74,7	129,72 ± 60,39	61,03 ± 61,84	33,33 ± 39,61	/	**F(1,53)=38.26, p<0.0001**	**F(1,53)=4.12, p=0.047**	NS

Effect of chronic stress on spatial and discrimination learning performances, and during an open-field test (mean ± SD) in quails with a high (E+) or a low (E-) emotionality. Bold values denote statistical significance at the p < 0.05 level. Only significant interactions are showed. NS = No significant interactions.

S2 Table. Results for cell proliferation, survival and neuronal differentiation and associated statistics

Brain structure/Marker	E-				E+				Statistics and p-values			
	Control		Stress		Control		Stress		E+ vs E-	Stress	Side	Interaction
**Hippocampus**	Left	Right	Left	Right	Left	Right	Left	Right				
PCNA density cells/mm²	23,6 ± 14,24	26 ± 22,27	15 ± 11,93	13,6 ± 11,631	28 ± 25,72	18 ± 24,58	5 ± 4,63	12,2 ± 8,28	t(1)=-0.757, p=0.45	**t(1)=-2.46, p=0.01**	t(1)=-0.091, p=0.92	NS
VZ BrdU density cells/mm²	53,71 ± 25,20	35,85 ± 20,6	61,12 ± 33,13	48,62 ± 28,46	59 ± 30,28	30,37 ± 21,32	46,87 ± 20,84	36,12 ± 24,67	t(1)=1.06, p=0.29	t(1)=0.49, p=0.62	**t(1)=-2.65, p=0.01**	NS
NVZ BrdU density cells/mm²	31,71 ± 9,08	33,28 ± 8,93	26 ± 6,71	24,5 ± 6,65	22 ± 7,80	23 ± 4,95	20,37 ± 7,36	19,25 ± 8,36	**t(1)=4.03, p<0.001**	**t(1)=-2.59, p=0.01**	t(1)=-0.03, p=0.97	NS
% BrdU+/DCX+	44,75 ± 8,04	53,37 ± 10,19	48,25 ± 14,43	51,12 ± 16,51	42,75 ± 13,41	41,62 ± 9,33	49,75 ± 11,88	44,25 ± 10,23	t(1)=1.59, p=0.11	t(1)=0.9, p=0.36	t(1)=0.4, p=0.68	NS
%BrdU+/NeuN+	0,5 ± 1,22	2,17 ± 2,56	1,57 ± 2,57	4,14 ± 3,80	0,57 ± 1,51	1 ± 2,64	1,25 ± 1,9	1,13 ± 1,55	t(1)=1.75, p=0.08	t(1)=1.41, p=0.16	t(1)=1.64, p=0.10	NS
**Median Striatum**												
PCNA density cells/mm²	5143 ± 1384,41	5515,2 ± 655,31	4587,4 ± 329,83	4704,4 ± 547,93	4915,6 ± 1142,4	4534 ± 1110,66	5114,8 ± 1042,75	4307,8 ± 1966,92	t(1)=-0.76, p=0.45	t(1)=-0.98, p=0.33	t(1)=-0.49, p=0.62	NS
BrdU density cells/mm²	65,58 ± 12,73	57,19 ± 11,62	69,34 ± 11,71	59,52 ± 17,95	67,42 ± 19,44	66,08 ± 18,26	72,32 ± 18,98	65,75 ± 17,61	t(1)=-1.23, p=0.22	t(1)=0.64, p=0.51	t(1)=-1.61, p=0.11	NS
% BrdU+/DCX+	53,14 ± 9,99	52,28 ± 11,51	54,5 ± 4,24	54,75 ± 10,84	56,25 ± 9,03	55 ± 8	59,5 ± 11,50	61,25 ± 11,80	t(1)=-1.76, p=0.07	t(1)=1.39, p=0.16	t(1)=0, p=1	NS
%BrdU+/NeuN+	8,85 ± 6,61	11,71 ± 3,35	10 ± 5,55	10,75 ± 3,37	8 ± 3,85	7,25 ± 3,19	11,75 ± 3,61	12,5 ± 3,16	t(1)=1.82, p=0.07	**t(1)=3.01, p=0.003**	t(1)=0.78, p=0.43	**Stress x Emotionality: t(1)=-2.17, p=0.03**

Effect of chronic stress on cell proliferation, survival and neuronal differentiation into the left and right hippocampus and median striatum (mean ± SD) in quails with a high (E+) or a low (E-) emotionality trait. VZ= ventricular zone; NVZ=non-ventricular zone; Bold values denote statistical significance at the p < 0.05 level. Only significant interactions are showed. NS = No significant interactions.
